# Sexually Dimorphic Gene Expression Associated with Growth and Reproduction of Tongue Sole (*Cynoglossus semilaevis*) Revealed by Brain Transcriptome Analysis

**DOI:** 10.3390/ijms17091402

**Published:** 2016-08-26

**Authors:** Pingping Wang, Min Zheng, Jian Liu, Yongzhuang Liu, Jianguo Lu, Xiaowen Sun

**Affiliations:** 1 Heilongjiang River Fisheries Research Institute, Chinese Academy of Fishery Sciences, Harbin 150070, China; wangpingping@hrfri.ac.cn (P.W.); mzz0003@tigermail.auburn.edu (M.Z.); sunxiaowen@hrfri.ac.cn (X.S.); 2 Environmental Engineering Program, Department of Civil Engineering, Auburn University, Auburn, AL 36849, USA; 3 School of Computer Science and Technology, Harbin Institute of Technology, Harbin 150001, China; jianliu@hit.edu.cn (J.L.); yongzhuang.liu@hit.edu.cn (Y.L.)

**Keywords:** sexually dimorphic, tongue sole, transcriptome analysis

## Abstract

In this study, we performed a comprehensive analysis of the transcriptome of one- and two-year-old male and female brains of *Cynoglossus semilaevis* by high-throughput Illumina sequencing. A total of 77,066 transcripts, corresponding to 21,475 unigenes, were obtained with a N50 value of 4349 bp. Of these unigenes, 33 genes were found to have significant differential expression and potentially associated with growth, from which 18 genes were down-regulated and 12 genes were up-regulated in two-year-old males, most of these genes had no significant differences in expression among one-year-old males and females and two-year-old females. A similar analysis was conducted to look for genes associated with reproduction; 25 genes were identified, among them, five genes were found to be down regulated and 20 genes up regulated in two-year-old males, again, most of the genes had no significant expression differences among the other three. The performance of up regulated genes in Gene Ontology (GO) and Kyoto Encyclopedia of Genes and Genomes (KEGG) pathway enrichment analysis was significantly different between two-year-old males and females. Males had a high gene expression in genetic information processing, while female’s highly expressed genes were mainly enriched on organismal systems. Our work identified a set of sex-biased genes potentially associated with growth and reproduction that might be the candidate factors affecting sexual dimorphism of tongue sole, laying the foundation to understand the complex process of sex determination of this economic valuable species.

## 1. Introduction

Tongue sole (*Cynoglossus semilaevis*) is a valuable marine fish in China, which is receiving more attention because of its good taste and nutritional value. However, the species is decreasing in number in the wild despite increasing market demand, which favors more production of the species [1]. Apart from its increasing economic value, it also has an intriguing reproductive biology. Genetically, tongue sole has ZW sex-determination system [2]. Males are the homogametic sex (ZZ), while females are the heterogametic sex (ZW). Male and female tongue sole are considerably different in size and growth rate: mature females are twice larger in length and six times greater in weight than their male counterparts [3]. Thus, understanding the underpinning of sexual dimorphism and sex determination in this species is essential for developing methods to boost its productivity to meet the aquaculture market demands.

Although sexual determination in some fish is partly determined by the environment [4,5,6], brain is still a major organ involved in fish growth and reproduction [7], and plays a key role in sexual dimorphism and sex determination, including the regulation of reproduction, maturation and sexual behavior in both sexes. The influence of brain on development is mainly mediated by the Brain–Pituitary–Gonadal (BPG) axis [8]. Gahr [9] found that male quail transplanted with female forebrain do not develop normal testes, indicating that a male brain is necessary for normal testes development. This demonstrates that sexual dimorphism in brain is not entirely determined by hormones secreted by the gonads, and brain develops differently between sexes even before the gonad development [10,11,12]. Therefore, understanding the mechanism of sexual dimorphism in brain is fundamental for enhancing our knowledge of development processes and exploring the genetic toolkit to maintain or change the difference between sexes.

Yang and colleagues [13] found that relatively small changes in gene expression might control functional sexual dimorphism in the brain, suggesting that differences in a few of those genes may become biological significant when interact with other genes in functional gene networks. Thus, uncovering the sex-biased genes are thought to be necessary when studying the mechanism of sexual dimorphism, and these genes may evolve differently under selection pressure, in turn, acting on the sexual phenotype [14].

For researchers, the major challenge is to explain the huge list of differentially expressed genes (DEGs) under certain conditions. A decade ago, automatic functional classification method using Gene Ontology (GO) was proposed to help study the function of these DEGs [15]. The first step is to group the list of DEGs into functional groups, which can offer insight into the biological mechanisms at the given condition. In addition, a well-characterized transcriptome is considered to be helpful to identify functional genes underlying the certain phenotypic variation in several ways.

Gonads, as the main sex organ, are often utilized to get a comprehensive overview of differentially expressed genes responsible for sexual dimorphism. Up to now, thousands of sex-biased genes have been identified from gonads in many species, including fruit fly [16], mouse [17] and some teleost, such as zebrafish [18], Nile tilapia [19], sharpsnout seabream [20], guppy [21], and yellow catfish [22]. However, sex-biased genes expressed in brain have been less well studied.

For the sexually dimorphic development of tongue sole, studies have mainly focused on the hormones expressed in brain known to influence the BPG-axis such as pituitary adenylate cyclase-activating polypeptide (PACAP) [23,24], growth hormone-releasing hormone (GHRH) [23,24] and growth hormone (GH) [25,26]. However, few studies have been published at the transcriptomic level to identify and characterize sex-biased genes related to growth and reproduction in tongue sole brain. Recently, the whole genome of tongue sole was published [2], enabling whole genome-based transcriptomic analyses.

In this study, we seek to identify the molecular mechanism underlying sexual dimorphism of the brain transcriptome of tongue sole. Fish used for this project were one- and two-year-old tongue sole. The average body weights of one-year-old male and female were 54 and 99 g, and the average body lengths were 21 and 25 cm, respectively. For two-year-old fish, the average weights were 337 and 1380 g, and average lengths were 27 and 42 cm, for male and female respectively. Females were much bigger than males. Two-year-old females were still immature, while males were mature. We used the RNA-Sequencing approach [27] aimed at identifying the differentially expressed genes of tongue sole. Brain transcriptomic profiles of one- and two-year-old males and females were analyzed and revealed a number of sexually differentially expressed genes potentially associated with growth and reproduction. These data will hopefully provide a useful genetic resource for further sex determination studies on tongue sole.

## 2. Results

### 2.1. Transcriptome Sequencing and Assembly

**Figure 1:**
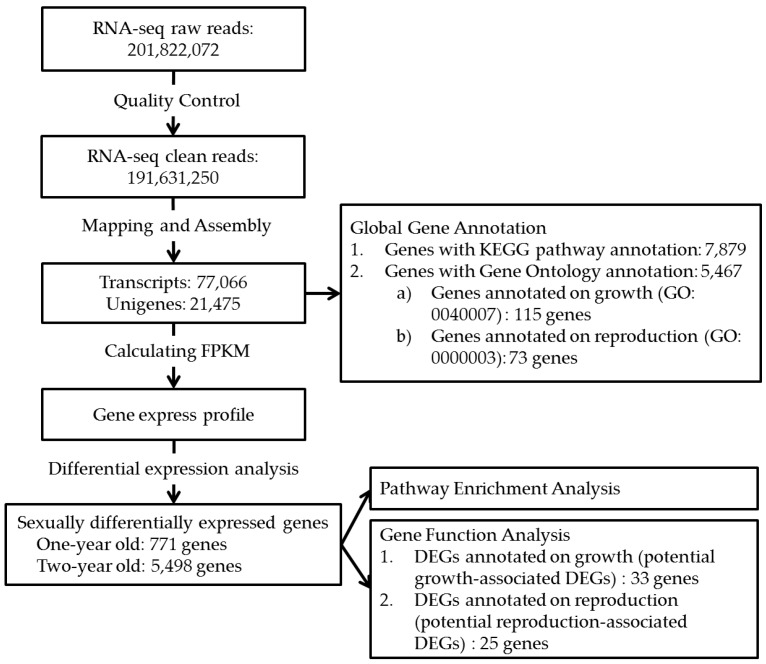
Work-flow of the transcriptome analysis of tongue sole.

To obtain comprehensive information of gene expression between sexes, we sequenced four transcriptomes derived from female and male brains at one and two-year-old tongue sole by using Illumina Hiseq2000 technology. A workflow with the transcriptome data analysis procedures is shown in Figure 1.

In total, 201,822,072 paired-end reads were generated, encompassing 20.2 Gb of sequence. Low quality reads and reads with ambiguous nucleotides were discarded, leaving 191,631,250 clean reads for transcriptome assembly and analysis. To assess our transcriptome assembly, we mapped our clean paired-end reads to the *C. semilaevis* whole genome. Approximately 84.2% of them exhibited significant hits to the genome. These reads were assembled into 77,066 transcripts in total, corresponding to 21,475 unigenes, with an average length of 3059 bp, the N50 value of 4349 bp and the N90 value of 1638 bp. Transcriptome data obtained from four samples has been uploaded to NCBI SRA site, with accession numbers of SRR2046146, SRR2046147, SRR2046153, and SRR2046154, respectively.

### 2.2. Gene Identification and Functional Annotation

All transcribed genes were functionally annotated by blasting the NCBI non-redundant database. A total of 10,185 genes were annotated with at least one significant hit (*E* value < 1 × 10^−5^). Of these genes, 5467 genes were further annotated by Gene Ontology (GO) with 5845 GO terms. A histogram of the number and percent of genes falling into the GO categories is given in Figure S1. The category of biological process contained the most genes, followed by molecular function and cellular component. Briefly, in biological process, cellular processes and metabolic processes were the most represented categories; with a total of 3553 genes assigned to cellular process (GO: 0009987, 64.6%) and 2756 genes to metabolic process (GO: 0008152, 50.2%). In the category with cellular component, cell (GO: 0005623, 59.2%) and organelle (GO: 0043226, 30.5%) were annotated with most abundant genes. Finally, in the category of molecular functions, binding and catalytic activity were two most frequent classes.

Functional pathway analysis based on the Kyoto Encyclopedia of Genes and Genomes (KEGG) database was also conducted as a complementary approach to categorize and annotate transcribed genes. Of the 21,475 transcribed genes, 7879 genes were assigned to KEGG orthologs, and then categorized into six functional groups (Table S1A). In short, of the genes with KEGG annotation, 1929 genes (22.5%) were assigned to metabolism, 1394 (17.7%) to genetic information processing, and 2774 genes (35.2%) were classified to environmental information processing. Cellular processes, organismal systems and human diseases contained 1883 (23.8%), 2944 (37.3%), and 2695 (34.2%) KEGG annotated genes, respectively.

### 2.3. Differentially Expressed Genes (DEGs) and Gene Enrichment Analysis

**Figure 2:**
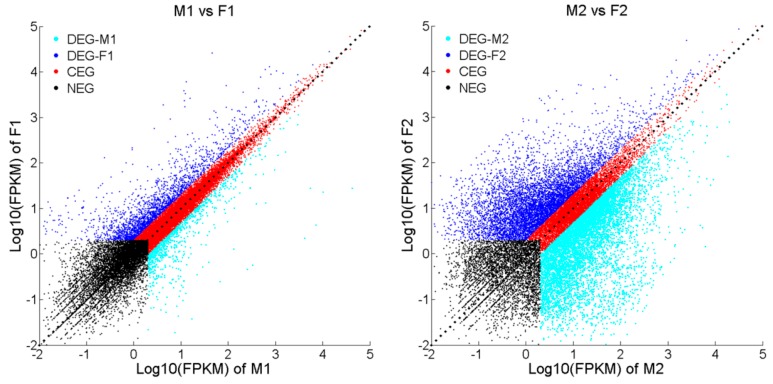
Gene expression profiling in brain tissue between male and female tongue sole at two developmental stages. Non-expressed genes (NEGs) marked as black dots. Differentially expressed genes (DEGs) marked as dark blue and light blue dots. Co-expressed genes (CEGs) marked as red dots.

Gene expression profiling of the male vs. female brain at two developmental stages are plotted in Figure 2. From the total of 21,475 transcribed genes, 771 genes were found to have sex-specific expression pattern in one-year-old fish, 469 of them were up-regulated in the male and the rest in the female. For the two-year-old fish, the number of DEGs (5498 genes) was about seven times greater than that in one-year-old fish. Among those DEGs, 3025 genes were found to be up-regulated in male, and the rest in female. More up-regulated genes were found in M2 than F2, which might be related to the maturation of males in this stage. Overall, co-expressed genes (CEGs) still made up the majority of all genes at both developmental stages. Transcriptome profiling and differentially expressed genes were listed in supplementary Table S2. DEGs, CEGs and NEGs between male and female tongue sole were shown in supplementary Table S4.

Gene enrichment analysis was conducted to further understand the biological functions of the DEGs. All DEGs were mapped to the terms of Gene Ontology and compared with the background of the whole transcriptome (Table 1). More DEGs in two-year-old fish were identified (711 up-regulated genes for male (M2), and 619 genes for female (F2)) involved in Gene Ontology enrichment analysis comparing to one-year-old fish (104 genes for male (M1), and 63 genes for female (F1)). DEGs were significantly enriched in several GO terms. In biological process, two-year-old male tongue sole (M2) had a strikingly greater gene expression in reproduction (20 genes), metabolic process (406 genes), cellular process (502 genes), anatomical structure formation (78 genes) and cellular component organization (134 genes), while the highly expressed genes of female (F2) were mainly enriched on multicellular organismal process (174 genes), localization (147 genes), establishment of localization (130 genes) and biological regulation (250 genes).

KEGG pathway enrichment analysis was also performed in Table S1B. Two-year-old male tongue sole had greater gene expression in genetic information processing, while female’s highly expressed genes were mainly enriched on organismal systems and human diseases pathway.

### 2.4. Genes with Sex-Biased Expression Potentially Associated with Growth

As a vital mariculture fish, growth differences between male and female tongue sole are attracting more and more attention of researchers. Two-year-old female tongue sole are bigger in size and have higher growth rates than their male counterparts. Strikingly different gene expression patterns were also observed in the brain of the two sexes (Figure 2).

Gene Ontology is known to describe the properties of genes and their products in any organism. Based on GO terms, we attempted to identify sex-biased genes related with sexually dimorphic phenotypic traits. Here, we mainly focus on detecting the sex-biased genes for growth and reproduction associated with GO terms.

According to GO annotation, 5467 genes were annotated by Gene Ontology. In biological process, 115 genes were assigned to growth (GO: 0040007) (Figure 1 and Figure S1). Thirty-three of the genes were sexually differentially expressed, which are called potential growth-associated DEGs in this study (Figure 1 and Table S3).

In two-year-old tongue sole, 30 differentially expressed genes were assigned to growth (Table 2), which accounted for 91% genes of potential growth-associated DEGs. The expression level of these genes in two development stages of tongue sole are plotted in Figure S2, and the significant analysis are listed in Table S3. Twelve genes were significantly highly expressed in M2 compared with F2 (Table 2 and Table S3A), including *h3f3b*, *hsc70*, *exosc9*, *vdac2*, *ppp2r1a*, etc. These genes were only highly expressed in M2, with no significant differences in gene expression among M1, F1 and F2 (Table S3A). Another 18 genes were found to be down regulated in two-year-old males compared to the other three samples (Table 2 and Table S3A), including *mdk*, *gap43*, *ptn*, *gja1*, *ninj2*, *cxcl12b*, *fgf12*, etc. These genes are mainly related to growth, fin regeneration and tissue regeneration. Significantly expressed DEGs were detected in all samples, but interestingly, most of these genes were only up or down regulated in M2 compared to the other samples.

### 2.5. Genes with Sex-Biased Expression Potentially Associated with Reproduction

Reproduction of tongue sole is another important focus in this study. According to GO annotation, 73 genes were assigned to reproduction (GO: 0000003) in biological process (Figure 1 and Figure S1). Twenty-five of them were differentially expressed between sexes, which were called potential reproduction-associated DEGs in this study (Table 3). The expression level of these genes in two development stages of tongue sole is plotted in Figure S3, and the statistical test analysis is listed in Table S3. For two-year-old fish, 20 genes were found to be up-regulated genes in males, which accounted for the majority of sex-biased genes influencing in reproduction (Table 3 and Table S3B). Those DEGs were only highly expressed in M2, and with no significant differences among the other three samples (Table S3B), which may be closely related to male development process. Nevertheless, only five genes were identified as female-biased genes (Table 3), and these genes were found to be down regulated in M2 compared to F2 (Figure S3B).

### 2.6. qPCR Validation of RNA-Seq Results

**Figure 3:**
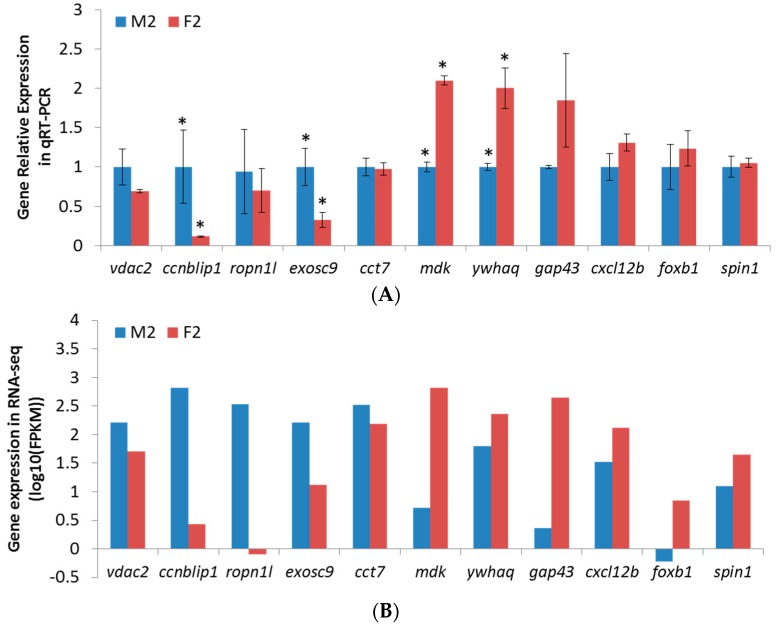
Brain transcriptome validation by qPCR using eleven selected genes: (**A**) Gene relative expression in qPCR where Error bars represent standard deviation of technical replicates and the expression differences between M2 and F2 were tested by two-tailed Student’s *t*-test, with 95% confidence level (*p* < 0.05) being labeled with an asterisk; and (**B**) gene expression in RNA-Seq.

In order to validate the expression pattern of DEGs identified by RNA-Sequencing, we randomly selected eleven genes from DEGs potentially associated with growth or reproduction (Table 2 and Table 3) for qPCR validation, including *vdac2*, *ccnblip1*, *ropn1l*, *exosc9*, cct7, *mdk*, *ywhaq*, *gap43*, *cxcl12b*, *foxb1* and *spin1*. M2 was used as the reference in 2^−∆∆*C*t^ method to calculate the relative gene expression level. Comparing the relative expression level of eleven selected genes, most results of qPCR were consistent with the results of RNA-Seq (Figure 3). The Pearson’s correlation of log_10_(fold-change) between qPCR and RNA-Seq was 0.80, indicating the accuracy and reliability of RNA-Seq based transcriptome analysis.

## 3. Discussion

This study conducted a transcriptome analysis of male and female brain tissues thereby providing the first assessment of the molecular mechanism underlying the reproductive development of tongue sole. We identified sex-biased genes and investigated up/down regulated DEGs with respect to two phenotypic traits (growth and reproduction). Finally, we validated eleven randomly selected potential growth or reproduction associated DEGs by qPCR, thereby supporting the reliability and accuracy of our transcriptome analysis.

### 3.1. The Brain Transcriptome of Tongue Sole

Brain transcriptome analysis was conducted on male and female tongue sole. The reason we chose one- and two-year-old individuals was two-fold. Firstly, males and females have significant differences in the age of maturity and body weight. Early sexual maturation of males was found at the age of nine months, while 21-month-old females were still immature [25]. The body weight of females was 3.28 times higher than males at 21 months [25]. We tried to capture the transcriptome differences between males and females at a stage with phenotypic divergence. Secondly, we looked for genes that contribute to sexual dimorphism, including upstream genes activity that might contribute to sex determination as well. We chose one- and two-year-old tongue sole for this research. Two-year-old males were mature, while females were still immature. The stage selection was appropriate and effective to characterize the sex-related transcriptomic expression profiles of tongue sole, since we identified a batch of differentially expressed genes in our assembled sequences.

### 3.2. Male and Female Brain Expression Pattern

Brain plays a central role in coordinating sexual function in mammals, and has remarkable plasticity in teleost [28]. Our results identified more genes to be over expressed in males than females, which is consistent with the findings on Manousaki’s work [20] on sharpsnout seabream, in which the authors identified a seven-fold increase of DEGs in two-year-old fish compared to one-year-old fish. More DEGs were detected in male tongue sole than female during two stages in our study.

### 3.3. Sexually Differentially Expressed Genes Potentially Associated with Growth

A number of genes involved in growth were found to be down regulated in two-year-old male brains. These included *mdk*, which encodes a member of a small family of secreted growth factors that binds heparin and promotes cell growth [29,30,31], migration, and angiogenesis [32,33]. It was also reported as a retinoic acid-responsive gene concerned with prenatal development and neuritis growth [34]. In mouse and human embryogenesis, *mdk* is widely expressed during mid gestation, but expression becomes undetectable in later stages [35,36]. In the brain of adult mice and rats, expression of *mdk* is reduced to undetectable levels [37]. *ptn*, often studied together with *mdk*, is a secreted growth factor that induces neurite outgrowth [38,39,40]. It is also claimed to play essential roles in female reproduction in mice [41]. One of these two genes deficient in mice will result in abnormal reproduction and development [41]. In rodents, these growth factors are highly expressed in early life and decrease to undetectable levels by adulthood in multiple organs [42]. *gap43*, a growth associated gene [43], plays a key role in nervous system [44] and it is related to nerve development, regeneration, and outgrowth. *ninj2* is known to play a role in nerve regeneration and in the formation and function of other tissues [45,46,47]. In this study, the decreased expression level of these genes in M2 might be related to the maturation of male in this stage with growth retardation. However, these genes keep a certain level during two stages in female while it was still growing, which indicated that they might be essential for female growth.

The up regulated genes potentially associated with growth in M2 included *h3f3b*, which is a replacement histone subtype in active genes [48], involved in DNA damage and cell cycle regulation [49,50]. *exosc9* encodes a component of the exosome complex with RNA degradation function [51], which prevents premature differentiation and promotes self-renewal by maintaining a low mRNA level of transcription factor *grhl3* [52]. These two genes both have a positive regulatory effect of cell growth. The expression level of *h3f3b* in two-year-old male (M2) was two times higher than other samples, and *exosc9* also shows strikingly higher expression level in two-year-old males (M2), which further suggests an enhanced rate for cell cycle regulation and enhanced self-renewal in the brain of male tongue sole.

### 3.4. Sexually Differentially Expressed Genes Potentially Associated with Reproduction

A few genes potentially associated with reproduction are discussed as follows. *ccnb1ip1*, also called as *hei10*, is a member of the E3 ubiquitin ligase family that acts as a limiting factor for crossing-over during meiosis [53] and involved in male gametogenesis and plays a role in cell cycle regulation [53,54]. When cells enter into mitosis, *ccnb1ip1* may interact with Cyclin B1 directly to control protein degradation in human [55], indicating its function of inhibiting cell growth.

*ropn1l* encodes a sperm protein of the ropporin family, which interacts with Protein Kinase A to regulate glycogen, sugar, and lipid metabolism [56]. Ropporin had been identified as a spermatogenic cell protein, but also has weak expression in normal non-testis tissues in latest studies, including brain tissue [57]. In Tonevitsky’s study [58], *ropn1l* had an increasing expression level in blood during the relaxation time after 30 min exercise of tested athletes. *ropn1l* was highly expressed in two-year-old male (M2), indicating its potential function in maturity male tongue sole. *cct4*, *cct5* and *cct7* are members of the chaperonin containing TCP1 complex (CCT), act as a molecular chaperone to assist the folding of proteins upon ATP hydrolysis [40,59,60], which are involved in binding of spermatozoa to the zona pellucida. The CCT complex is a large multi-subunit complex that assists the folding of a variety of proteins, including several tubulin and actin proteins [61,62] and it plays a key role in cell cycle regulation, cytoskeleton assembly and cell division [61]. Grantham [61] indicated that full CCT activity is essential for normal cell growth and division in mammalian cell. The functional significance of the increased *cct4*, *cct5* and *cct7* expression was unclear, but may have a possible relationship with the quicker maturation in males compared to females.

Other genes found in our research that might have significance but still unknown roles in the brain associated with growth and reproduction. Despite the relatively low number, these genes may become biological significant when interacting with the other genes in functional gene networks controlling and maintaining reproduction and development of tongue sole.

## 4. Materials and Methods

### 4.1. Experimental Fish and Sample Collection

All procedures involving sample handling and treatment used in this study were approved by the Animal Care and Use Committee at Heilongjiang River Fisheries Research Institute (ACUC-HRFRI). All experiments were conducted in accordance with the relevant guidelines and regulations. The fish used in this study included 20 male tongue sole (10 one-year-old juveniles and 10 two-year-old adults) and 20 female tongue sole (10 one-year-old juveniles and 10 two-year-old adults). The average body weights of one-year-old males and females were 54 and 99 g, and the average body lengths were 21 and 25 cm, respectively. For two-year-old fish, the average weights were 337 and 1380 g, and average lengths were 27 and 42 cm, for males and females, respectively. Females were much bigger than males. Two-year-old females were still immature, while males were mature. All forty fish came from one full-sib family in National Fish Original Species Farm at Tianjin, China. One-year-old fish were collected on 10 August 2013 and two-year-old on 12 August 2014. The sex of the fish was confirmed anatomically. Tissues of the whole brain from one- and two-year-old fish were collected and stored in 1.5 mL RNAlater tube (QIAGEN, Hilden, Germany), and then stored at −80 °C freezer for subsequent RNA extraction.

### 4.2. RNA Isolation

Brain samples were taken out of the −80 °C freezer and homogenerated with a TissueRuptor (QIAGEN) to a fine solution and then treated with RNase free DNase I (QIAGEN) for high RNA quality and yield. Total RNA were extracted from each sample using the RNeasy Lipid Tissue Kit (QIAGEN) according to the manufacturer’s instructions. The quality and quantity of RNA were examined by Thermo ScientificTM NanoDropTM 8000 Spectrophotometer (Thermo Scientific, Wilmington, DE, USA) and Agilent 2100 Bioanalyzer (Agilent Technologies, Santa Clara, CA, USA). Only RNA with OD260/280 ≥ 1.8 and RNA integrity number (RIN) ≥ 7 was stored for the following library construction and sequencing. Equal quantities of high quality RNA from each brain sample were pooled together for subsequent cDNA synthesis and sequencing.

### 4.3. cDNA Library Construction and Illumina Sequencing

Four RNA-Seq libraries were prepared and sequenced by BerryGenomics sequencing company (Beijing, China). cDNA library was constructed following the protocol of Illumina TrueSeq RNA Sample Prep Kit (Illumina, San Diego, CA, USA). The library was sequenced using Illumina HiSeq 2000 platform. In total, 100 bp paired-end reads with an insert size of 330 were obtained. The clean reads from the four transcriptomes were obtained by filtering out low quality reads (reads with quality value of ≤20) and adaptor-only reads from the raw data.

### 4.4. Sequence Assembly and Analysis

The quality control of RNA-Seq data was conducted by NGS QC Toolkit [63] with default parameters. Clean paired-end reads were aligned to the genome of tongue sole [2] using TopHat [64]. Then the mapping files were analyzed using Cufflinks [65] to assemble the reads into transcripts for each dataset. Complete transcripts were obtained by merging the assemblies of all datasets using Cuffmerge. Duplicate sequences were removed by cd-hit [66].

### 4.5. Functional Annotation and Ontology

All expressed genes were aligned to NCBI non-redundant (NR) protein database by using BLASTP with *E*-value < 1 × 10^−5^. With the result of BLASTP, Gene ontology (GO) [67] annotation was performed using Blast2GO [68]. The top two GO terms associated with transcripts were retrieved, and then the annotation genes were grouped into three categories, biological process, molecular function, and cellular components. Genes annotated on growth (GO: 0040007: BP) and reproduction (GO: 0000003: BP) were selected as potential growth- and reproduction-associated genes, respectively. Pathways from Kyoto Encyclopedia of Genes and Genomes (KEGG) [69] were assigned with differential expressed genes by using the online KEGG Automatic Annotation Server (KAAS) [70].

### 4.6. Identification of Differentially Expressed Genes (DEGs) and Pathway Enrichment Analysis

FPKM (Fragments Per Kilobase of exon per Million fragments mapped reads) is widely used in RNA-Sequencing to represent the gene expression level [71]. The FPKM is calculated by Cuffdiff [72] method. Then, DEGseq [73] was used to identify sexually differentially expressed genes (DEGs) based on the MARS model (MA-plot-based method with Random Sampling model). Genes with “*p*-value < 0.001” were selected as DEGs. False discovery rate (FDR) was widely set at 0.05 to determine the threshold for the p-value. Sex-biased genes were strictly identified from DEGs with at least 2-fold difference between genders (|log_2_(FRKM_ZZ/FPKM_ZW)| ≥ 1). Genes meeting “FPKM_ZZ < 2 and FPKM_ZW < 2” statistical criteria were grouped as non-expressed genes (NEGs). All remaining genes were classified as co-expressed genes (CEGs). This way, all transcribed genes were classified into three groups: DEGs, CEGs and NEGs.

Fisher’s exact test was conducted to further detect functional DEGs for RNA-Seq data [74,75]. The right-tailed Fisher’s exact test was used to identify the enriched GO terms or KEGG categories. All transcribed genes were divided into two sets, differentially expressed genes (DEGs) and non-DEGs. Fisher’s exact test was calculated based on a 2 × 2 contingency table (Table 4). The two columns of the table were separated by DEGs and non-DEGs. The two rows were separated by the given category A and other categories.

The Fisher’s exact test *p*-value for each category was calculated in the following formula.

$$p=\frac{C_{a+b}^{a}C_{c+d}^{c}}{C_{a+b+c+d}^{a+c}}=\frac{\left(a+b\right)!\left(c+d\right)!\left(a+c\right)!\left(b+d\right)!}{\left(a+b+c+d\right)!a!b!c!d!}$$

*p*-Value ≤ 0.05 were selected as a threshold to determine the significant enriched GO terms or KEGG pathways.

### 4.7. Experimental Validation by qPCR

Sexually differentially expressed genes were validated using quantitative real-time PCR (qPCR). Brain samples were dissected from three male and three female tongue sole at both one and two years of age. Total RNA was extracted from each brain sample and reverse-transcribed using MMLV reverse transcriptase (Invitrogen, Carlsbad, CA, USA). Primers used in qPCR were designed with the help of Primer Premier 6.0. Quantitative real-time PCR was performed using the SYBR Green I Master Mix (TaKaRa, Dalian, China) in Applied Biosystems Prism 7500-fast real-time PCR system. The PCR reaction conditions were as follows: 95 °C for 5 min, followed by 40 amplification cycles of 95 °C for 15 s and 60 °C for 30 s. β-Actin had been validated as a useful internal control for gene expression studies using quantitative real-time PCR in tongue sole [24,76], and was used as the reference gene to normalize the mRNA expression levels of all the validated genes in this study. The sequence of beta-actin gene and primers used in this study were referred from Chen’s study [2]. The fold change of each gene was calculated by the relative quantification (2^−∆∆*C*t^) method [77]. qPCR analysis was repeated three times to confirm gene expression patterns. Expression differences between samples were tested by two-tailed Student’s *t*-test, and 95% confidence level (*p* < 0.05) was selected to determine the differentially expressed genes.

## 5. Conclusions

In this study, we performed a comprehensive analysis of the transcriptome of one- and two-year-old male and female brains of *Cynoglossus semilaevis* by high-throughput Illumina sequencing. A total of 21,475 unigenes were obtained by transcriptome analysis. Differential expression analysis combined with gene enrichment analysis revealed a number of potential growth and reproduction associated genes. Most of these genes are only up or down regulated in two-year-old males, and had no significant differences in expression among one-year-old males and females and two-year-old females. Potential growth and reproduction associated genes in our work might be the candidate factors affecting sexual dimorphism in tongue sole, laying the foundation to understand the complex process of development and sex determination of this economic valuable species.

## Figures and Tables

**Table 1:** The Gene ontology enrichment analysis of differential expressed genes. For Fisher’s exact test, *p*-value ≤ 0.05 is labeled as yellow, and *p*-value ≤ 0.01 is labeled as red.

GO Category	GO Term	Total No. of Genes	M1 vs. F1		M2 vs. F2	
			M1	F1	M2	F2
Cellular Component	GO:0005576 extracellular region	310	10	9	40	49
	GO:0005623 cell	3238	69	38	425	400
	GO:0019012 virion	11	3	0	5	2
	GO:0031974 membrane-enclosed lumen	408	8	2	83	31
	GO:0031975 envelope	105	7	0	22	18
	GO:0032991 macromolecular complex	1014	34	26	175	151
	GO:0043226 organelle	1669	35	28	282	212
	GO:0044421 extracellular region part	214	3	4	34	33
	GO:0044422 organelle part	1008	29	15	193	127
	GO:0044423 virion part	11	3	0	5	2
	GO:0044456 synapse part	75	3	1	3	23
	GO:0044464 cell part	3238	69	38	425	400
	GO:0045202 synapse	102	6	1	5	28
Molecular Function	GO:0003824 catalytic activity	2105	34	13	298	210
	GO:0005198 structural molecule activity	112	9	18	20	42
	GO:0005215 transporter activity	393	19	5	30	68
	GO:0005488 binding	2780	53	31	380	318
	GO:0009055 electron carrier activity	6	0	0	1	1
	GO:0015457 auxiliary transport protein activity	7	0	1	1	2
	GO:0016209 antioxidant activity	14	0	0	1	2
	GO:0030234 enzyme regulator activity	200	0	4	22	35
	GO:0030528 transcription regulator activity	188	2	1	14	14
	GO:0042056 chemoattractant activity	2	0	0	0	1
	GO:0045182 translation regulator activity	26	1	0	8	2
	GO:0045499 chemorepellent activity	1	0	0	0	0
	GO:0060089 molecular transducer activity	430	3	2	19	35
Biological Process	GO:0000003 reproduction	73	0	0	20	5
	GO:0001906 cell killing	3	0	0	0	1
	GO:0002376 immune system process	189	2	1	28	26
	GO:0008152 metabolic process	2756	46	37	406	297
	GO:0008371 obsolete biological process	58	4	0	4	8
	GO:0009987 cellular process	3553	71	48	502	403
	GO:0010926 anatomical structure formation	378	8	8	78	43
	GO:0016032 viral reproduction	30	1	1	8	8
	GO:0016043 cellular component organization	652	15	8	134	77
	GO:0016265 death	169	2	2	32	14
	GO:0022414 reproductive process	72	1	1	19	10
	GO:0022610 biological adhesion	113	1	0	13	15
	GO:0032501 multicellular organismal process	1315	34	17	151	174
	GO:0032502 developmental process	1124	26	14	147	129
	GO:0040007 growth	115	5	3	12	18
	GO:0040011 locomotion	152	1	1	18	22
	GO:0043473 pigmentation	1784	33	26	198	216
	GO:0044085 cellular component biogenesis	327	11	22	75	58
	GO:0048511 rhythmic process	11	0	0	0	1
	GO:0050896 response to stimulus	606	19	9	81	83
	GO:0051179 localization	955	30	11	120	147
	GO:0051234 establishment of localization	790	30	10	100	130
	GO:0051704 multi-organism process	55	2	1	11	11
	GO:0065007 biological regulation	1927	38	29	219	250
All genes annotated by GO		5467	104	63	711	619

**Table 2:** Differentially expressed genes (DEGs) in two-year-old tongue sole potentially associated with growth.

Gene	FPKM		*p*-Value	Annotation	GO Function
	M2	F2	M2 vs. F2		
**DEGs Up Regulated in M2**					
*h3f3b*	670.6	193.2	1.9E-56	Histone H3.3	Positive regulation of cell growth (GO:0030307)
*hsc70*	247.9	0.1	5.5E-34	Heat shock cognate 70 kDa protein	Fin regeneration (GO:0031101)
*exosc9*	164.6	13.2	1.7E-32	Exosome complex component RRP45	Positive regulation of cell growth (GO:0030307)
*vdac2*	164.4	51.1	7.9E-14	Voltage-dependent anion-selective channel protein 2	Fin regeneration (GO:0031101)
*ppp2r1a*	237.7	108.1	9.4E-11	Serine/threonine-protein phosphatase 2A 65 kDa regulatory subunit A β isoform	Growth (GO:0040007)
*timm50*	44.6	12.1	2.5E-05	Mitochondrial import inner membrane translocase subunit TIM50	Growth (GO:0040007)
*bmp2*	30.6	6.1	5.3E-05	Bone morphogenetic protein 2	Growth (GO:0040007)
*ascl1*	17.0	1.1	9.1E-05	Achaete-scute homolog 1	Sensory epithelium regeneration (GO:0070654)
*suv420h1*	31.8	7.3	1.1E-04	Histone-lysine *N*-methyltransferase SUV420H1	Regulation of multicellular organism growth (GO:0040014)
*orc1*	15.7	1.1	1.8E-04	Origin recognition complex subunit 1	Regulation of multicellular organism growth (GO:0040014)
*nenf*	26.8	5.7	2.2E-04	Neudesin neurotrophic factor	Growth (GO:0040007)
*dnajc2*	42.6	16.0	9.6E-04	dnaJ homolog subfamily C member 2	Negative regulation of cell growth (GO:0030308)
**DEGs Down Regulated in M2**					
*mdk*	5.3	667.8	1.7E-157	Midkine	Growth (GO:0040007)
*gap43*	2.3	442.4	2.3E-100	Growth associated protein 43	Tissue regeneration (GO:0042246)
*ptn*	30.3	287.1	3.1E-56	Pleiotrophin	Growth (GO:0040007)
*gja1*	1.9	222.0	6.1E-54	Gap junction α-1 protein	Fin regeneration (GO:0031101)
*ninj2*	55.5	255.5	1.1E-34	Ninjurin-2	Tissue regeneration (GO:0042246)
*cxcl12b*	33.3	131.8	1.1E-16	Stromal cell-derived factor 1 precursor	Fin regeneration (GO:0031101)
*fgf12*	2.0	56.1	5.0E-15	Fibroblast growth factor 12	Growth (GO:0040007)
*gcfc2*	23.4	102.5	2.3E-14	GC-rich sequence DNA-binding factor 2	Fin regeneration (GO:0031101)
*sox2*	0.7	38.5	5.3E-11	Transcription factor SOX-2	Fin regeneration (GO:0031101)
*fyna*	2.8	38.2	1.0E-09	Tyrosine-protein kinase fyna	Fin regeneration (GO:0031101)
*epha4*	1.7	34.5	1.9E-09	Ephrin type-A receptor 4	Negative regulation of collateral sprouting of intact axon in response to injury (GO:0048685)
*bdnf*	1.9	29.3	6.6E-08	Brain-derived neurotrophic factor	Growth (GO:0040007)
*cdh2*	0.6	24.8	1.5E-07	cadherin-2-like	Tissue regeneration (GO:0042246)
*rerg*	3.0	29.9	2.6E-07	Ras-related and estrogen-regulated growth inhibitor	Negative regulation of cell growth (GO:0030308)
*ctgf*	2.5	22.9	9.4E-06	Connective tissue growth factor	Regulation of cell growth (GO:0001558)
*igfbp1*	0.2	17.9	9.8E-06	Insulin-like growth factor-binding protein 1	Regulation of cell growth (GO:0001558)
*klf6*	2.6	22.5	1.3E-05	Krueppel-like factor 6	Organ growth (GO:0035265)
*sptbn1*	7.0	27.5	1.7E-04	Spectrin β chain, non-erythrocytic 1	Positive regulation of multicellular organism growth (GO:0040018)

**Table 3:** Differentially expressed genes (DEGs) in two-year-old tongue sole potentially associated with reproduction.

Gene	FPKM		*p*-Value	Annotation	GO Function
	M2	F2	M2 vs. F2		
**pleaDEGs Up Regulated in M2**					
*ccnb1ip1*	661.4	2.7	3.6E-138	E3 ubiquitin-protein ligase CCNB1IP1	Reproductive cellular process (GO:0048610)
*rsph1*	516.7	5.7	1.4E-118	Radial spoke head 1 homolog	Reproduction (GO:0000003)
*ropn1l*	343.4	0.8	1.5E-66	Ropporin-1-like protein	Spermatid development (GO:0007286)
*alkbh5*	180.5	17.9	4.5E-33	RNA demethylase ALKBH5	Spermatogenesis (GO:0007283)
*henmt1*	150.3	0.0	2.4E-29	Small RNA 2’-*O*-methyltransferase	Oocyte development (GO:0048599)
*cct5*	358.3	136.8	3.4E-21	Chaperonin containing T-complex polypeptide 5	Binding of sperm to zona pellucida (GO:0007339)
*ovol1*	112.1	0.1	3.2E-19	Putative transcription factor Ovo-like 1	Spermatogenesis (GO:0007283)
*klhl10*	54.7	0.0	7.0E-14	Kelch-like protein 10	Spermatid development (GO:0007286)
*cct7*	329.3	153.7	1.4E-13	Cct7 protein	Binding of sperm to zona pellucida (GO:0007339)
*cct4*	305.8	147.9	8.8E-12	Cct4 protein	Binding of sperm to zona pellucida (GO:0007339)
*fem1b*	44.6	3.0	2.5E-10	Protein fem-1 homolog B-like	Epithelial cell maturation involved in prostate gland development (GO:0060743)
*sf1*	103.4	33.7	9.2E-09	Splicing factor 1	Leydig cell differentiation (GO:0033327)
*styx*	69.8	20.2	3.6E-07	Serine/threonine/tyrosine-interacting protein	Spermatogenesis (GO:0007283)
*ptbp1*	45.3	9.4	1.2E-06	Polypyrimidine tract-binding protein 1-like	Positive regulation of cortical granule exocytosis by elevation of cytosolic calcium ion concentration (GO:0060472)
*tbp*	41.8	9.0	4.4E-06	TATA box binding protein	Spermatogenesis (GO:0007283)
*pld6*	24.9	2.6	1.1E-05	Phospholipase D family member 6	P granule organization (GO:0030719)
*polr2h*	50.9	15.8	3.2E-05	DNA-directed RNA polymerases I%2C II%2C and III subunit RPABC3	Positive regulation of viral transcription (GO:0050434)
*pacrg*	38.8	10.5	7.8E-05	Parkin coregulated gene protein	Spermatid development (GO:0007286)
*tceb1*	137.6	75.3	1.2E-04	Transcription elongation factor B polypeptide 1	Positive regulation of viral transcription (GO:0050434)
*tdrd9*	13.3	0.6	2.9E-04	Putative ATP-dependent RNA helicase TDRD9	Fertilization (GO:0009566); DNA methylation during gametogenesis (GO:0043046)
**DEGs Down Regulated in M2**					
*ywhaq*	63.5	229.4	1.1E-25	YWHAQ protein	Germarium-derived oocyte fate determination (GO:0007294)
*cxcl12b*	33.3	131.8	1.1E-16	Stromal cell-derived factor 1-like	Germ cell migration (GO:0008354)
*spin1*	12.4	44.6	4.0E-06	Spindlin-1-like	Gamete generation (GO:0007276)
*stau2*	41.7	80.2	1.1E-04	Double-stranded RNA-binding protein Staufen homolog 2	Germ cell migration (GO:0008354)
*sptbn1*	7.0	27.5	1.7E-04	Spectrin β chain, non-erythrocytic 1	Fertilization (GO:0009566)

**Table 4:** 2 × 2 contingency table for Fisher’s exact test.

Gene Annotation	DEGs	Non-DEGs	All Transcribed Genes
Category A	a	b	a + b
Other categories	c	d	c + d
All categories	a + c	b + d	a + b + c + d

“a” represented the No. of DEGs annotated on category A; “b” represented the No. of non-DEGs annotated on category A; “c” represented the No. of DEGs annotated on the other categories except category A; and “d” represented the No. of non-DEGs annotated on the other categories except category A.

## Supplementary Materials

Supplementary materials can be found at www.mdpi.com/1422-0067/17/9/1402/s1.

## Abbreviations

GO	Gene Ontology
DEG	Differentially Expressed Gene
NEG	Non-Expressed Gene
CEG	Co-Expressed Gene
FPKM	Fragments Per Kilobase of exon per Million fragments mapped reads
qPCR	quantitative real-time PCR
