# Supplementary material for: Sexually Dimorphic Gene Expression Associated with Growth and Reproduction of Tongue Sole (Cynoglossus semilaevis) Revealed by Brain Transcriptome Analysis
Source: Int J Mol Sci. 2016 Aug 26;17(9):1402. doi: 10.3390/ijms17091402 (PMC5037682; doi:10.3390/ijms17091402)
Supplement: Supplementary file 1 [file ijms-17-01402-s001.zip › ijms-126499-Supplementary Materials/ijms-126499-Supplementary.pdf]

Supplementay Materials: Sexually Dimorphic Gene Expression Associated with Growth and Reproduction of Tongue Sole (*Cynoglossus semilaevis*) Revealed by Brain Transcriptome Analysis

Pingping Wang, Min Zheng, Jian Liu, Yongzhuang Liu, Jianguo Lu and Xiaowen Sun

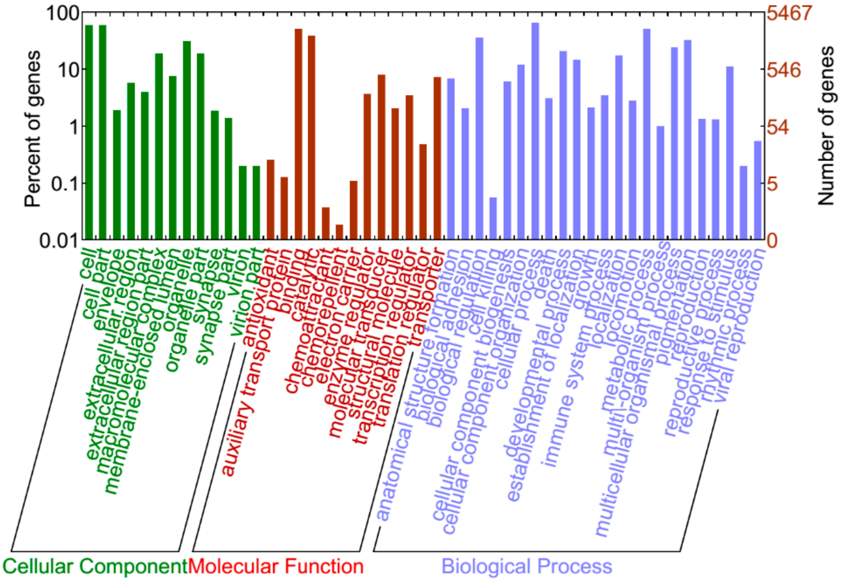

Figure S1. Gene ontology (GO) category distribution of tongue sole transcribed genes. 21,475 unigenes were annotated in 3 categories: cellular component, molecular function, and biological processes. In biological process, 115 genes were annotated on growth, and 73 genes were annotated on reproduction.

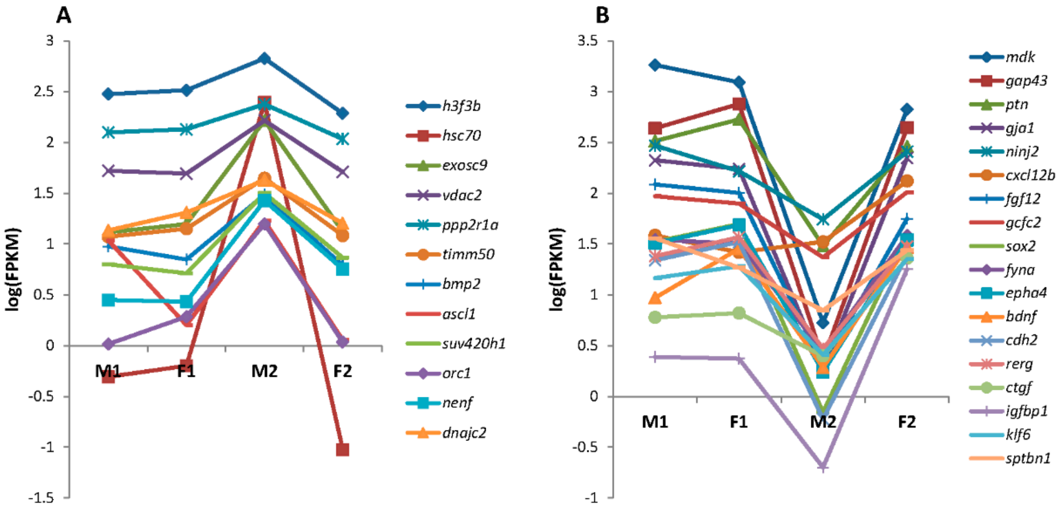

Figure S2. The expression level of up/down-regulated genes associated with growth for two developmental stages of male and female tongue sole. (**A**) The differentially expressed genes identified associated with growth, but only up regulated in M2; (**B**) The differentially expressed genes identified associated with growth, but only down regulated in M2.

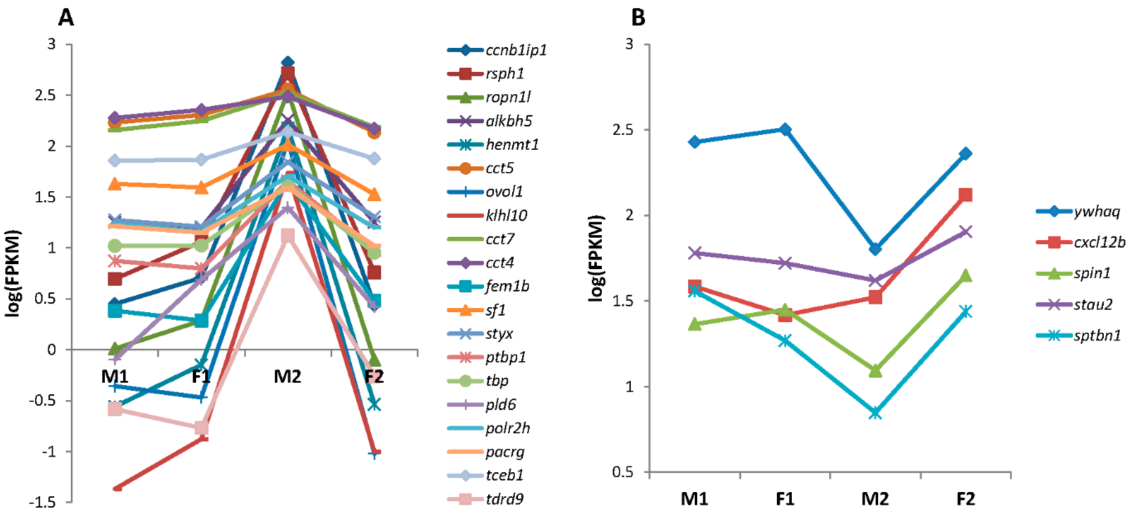

Figure S3. The expression level of up/down-regulated genes associated with reproduction for two developmental stages of male and female tongue sole. (**A**) The differentially expressed genes identified associated with reproduction, but only up regulated in M2; (**B**) The differentially expressed genes identified associated with reproduction, but only down regulated in M2.
